# Case Report: Talar Neck Fracture

**DOI:** 10.21980/J8FP75

**Published:** 2020-07-15

**Authors:** Wilson Frasca, Nhan Do

**Affiliations:** * Riverside Community Hospital/University of California Riverside, Department of Emergency Medicine, Riverside, CA

## Abstract

This report demonstrates a case of a severe talar neck fracture. Although rare, talar neck fractures have a high potential for morbidity. Typically caused by a high energy injury, this patient’s mechanism of injury was relatively minor, and presentation was not immediately concerning for such a severe fracture. Initial x-rays provided a gross demonstration of the fracture, but a CT scan was obtained to better evaluate the bone fragments and associated tarsal dislocations. The patient was treated symptomatically and splinted in the emergency department, underwent open reduction and internal fixation the next morning, and was discharged a day later. The patient’s severe injury was identified and received definitive treatment to help avoid potentially devastating complications.

**Topics:**

Orthopedics, trauma, talar neck fracture.

**Figure f1-jetem-5-3-v7:**
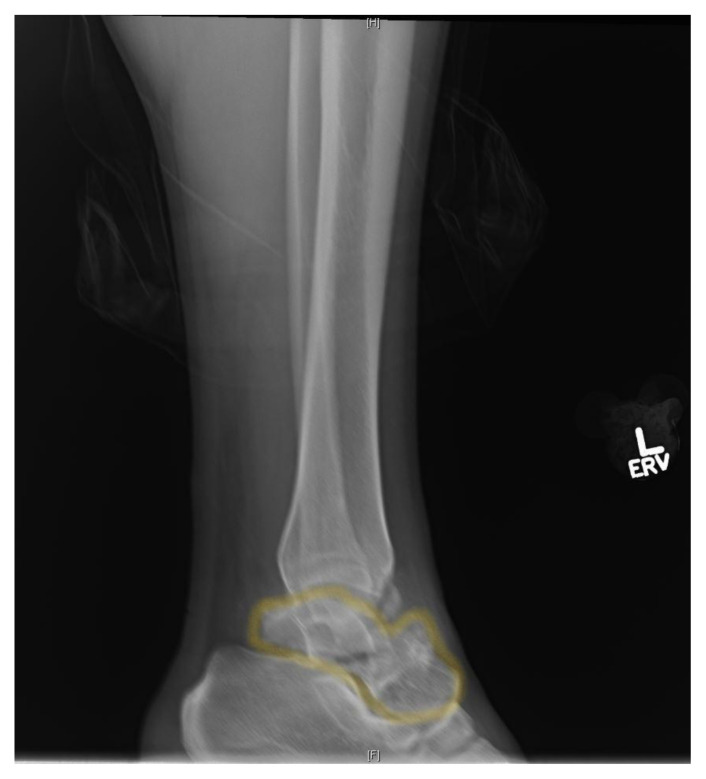


**Figure f2-jetem-5-3-v7:**
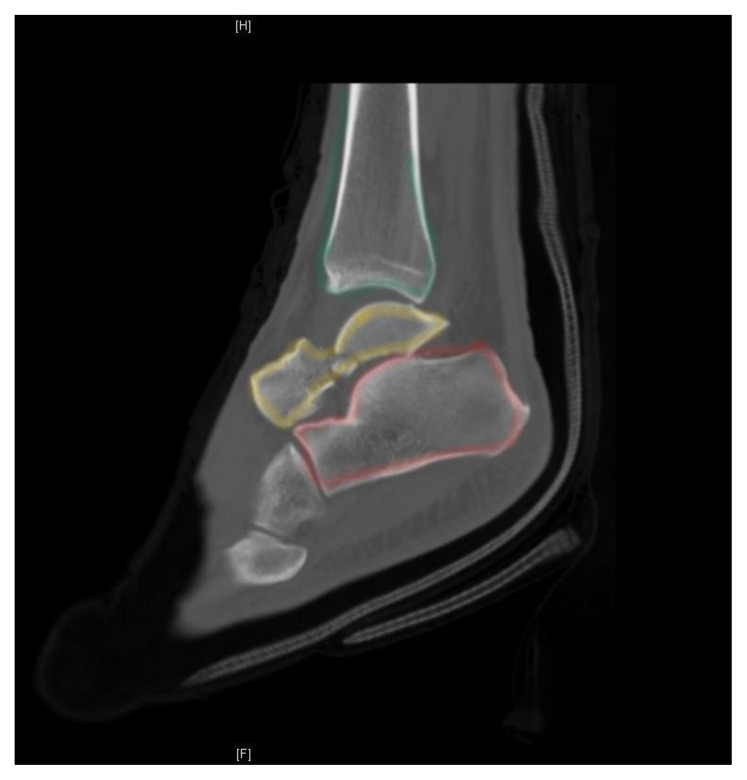


**Figure f3-jetem-5-3-v7:**
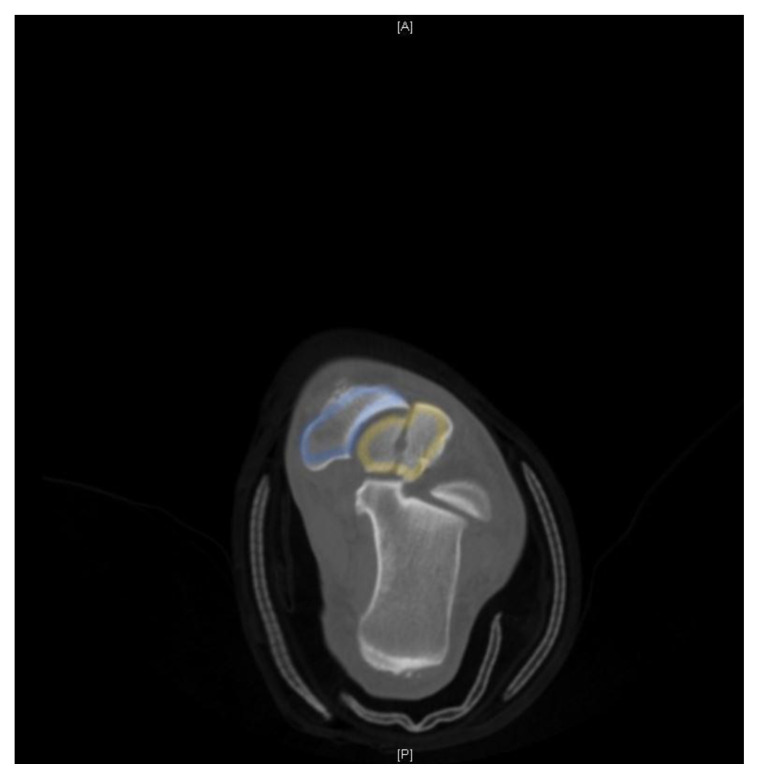


## Introduction

[Fig f1-jetem-5-3-v7][Fig f2-jetem-5-3-v7][Fig f3-jetem-5-3-v7]Talus neck fractures are uncommon, and are frequently reported to account for less than 1% of all fractures.^1^ Though rare, talar neck fractures are associated with significant complications and morbidity, most notably osteonecrosis. This injury usually involves a high energy mechanism. X-ray remains a staple of diagnosing orthopedic injuries. Computed tomography (CT) is not necessary to make the diagnosis in cases of severe fracture such as this one, but the orthopedic surgeon may request it to help guide repair. CT should be ordered by the emergency physician if there is suspicion for a subtle fracture not seen on x-ray. This took place at a capable community hospital with readily available orthopedic consultants. This case demonstrates a severe injury due to a relatively minor mechanism of injury in an otherwise healthy individual. It also highlights the importance of proper imaging and prompt orthopedic consultation and intervention to help reduce potentially severe complications.

## Presenting concerns and clinical findings

A 32-year-old male presented to the emergency department (ED) with a complaint of left ankle pain. The patient jumped over a fence at a community baseball field landing on his feet, causing immediate pain in his left ankle. On arrival to the ED he had moderate pain, did not appear uncomfortable, and was talking cheerfully with staff and his spouse. He was unable to bear weight, and was noted to have swelling and painful range of motion of the left ankle. The foot appeared to be diffusely swollen, but was not significantly deformed. Motor function, sensation, and pulses were not affected.

## Patient Course

The patient was treated in the emergency department with intravenous hydromorphone. Closed reduction performed by the emergency physician was partially successful and the leg was immobilized with a three-sided splint using prefabricated fiberglass materials. An orthopedic surgeon was consulted, and the patient was admitted for urgent surgery. The patient underwent successful open reduction and internal fixation of the talus with near-anatomical realignment. He was discharged the next day with non-weight bearing status and outpatient orthopedic follow-up.

## Significant findings

X-ray of the left ankle demonstrated a comminuted talar neck fracture with posterior subluxation of the subtalar joint (talus in yellow). CT scan of the ankle better demonstrated a severely comminuted fracture of the talar neck, body, and head. There were also tibiotalar and subtalar dislocations. CT imaging better shows talar neck fracture (yellow), as well as dissociation from the distal tibia (green) and calcaneus (red). The transverse talar head fracture (yellow) and 7 millimeter dislocation from the navicular bone (blue) is also shown.

## Discussion

The talus is the second largest tarsal bone, articulating with the distal tibia and fibula to form the ankle joint.^2^ The talus has no muscle attachment and is held in place by ligaments and articular interfaces.^2^,^3^ The neck is the area of the talus most susceptible to fracture, and accounts for nearly half of all talus fractures.^2^ The talar neck is particularly susceptible to fracture due to its relatively decreased amount of trabecular bone and its abrupt change in trabecular orientation.^1^ While talus fractures represent less than 1% of all lower extremity fractures, they are associated with significant morbidity, most notably posttraumatic arthritis, malunion, and avascular necrosis.^1^,^2^,^3^,^4^,^5^,^6^,^7^

Fractures of the talar neck are categorized using the Hawkins Classification:^3^

- Type 1: Nondisplaced fracture
- Type 2: Displaced fracture with subtalar dislocation/subluxation
- Type 3: Displaced fracture with subtalar and tibiotalar dislocation/subluxation
- Type 4: Type 3 fracture with additional talonavicular dislocation/subluxation

This classification has been shown to be associated directly with complications, with osteonecrosis seen in nearly 100% of Hawkins Type 4 fractures.^1^,^4^,^5^ The talus blood supply has generally been described as tenuous and variable, which is why disruption with severe fractures may lead to avascular necrosis.^1^,^2^,^3^ More recent studies have shed further light on this matter using gadolinium-enhanced magnetic resonance angiography. A substantial portion of the blood supply enters posteriorly, rather than retrograde as previously described, which may help explain why not all talar neck fractures result in osteonecrosis.^6^

Fractures of the talar neck and body are associated with high energy mechanisms, such as a fall from height or motor vehicle accidents.^2^,^4^,^5^ These mechanisms of injury cause axial loading with severe dorsiflexion of the ankle, forcing the talus to impinge on the distal tibia. Based on the mechanism, malleolar injuries are possible. In cases of falls from significant heights, spinal column injuries may be considered as well. Patients typically present with pain and hindfoot swelling, rather than deformity.^4^

Three-view ankle x-rays should be obtained. Prompt closed reduction under procedural sedation should be performed for displaced talar neck fractures, dislocations, or if there is concern for neurovascular compromise.^2^ When suspecting a talus injury, computed tomography is the imaging modality of choice to fully evaluate for fractures.^2^

This case demonstrates the importance of appropriate imaging modalities and prompt surgical evaluation for talar neck fractures. Although the initial x-ray clearly showed a talar neck fracture, the degree of comminution and associated dislocations were better appreciated on CT. This additional information helps guide surgical management, avoids undertreatment, and minimizes poor outcomes for the patient.^7^

## Supplementary Information












