# Supplementary figures and images for: Case Report: Talar Neck Fracture
Source: J Educ Teach Emerg Med. 2020 Jul 15;5(3):V7–9. doi: 10.21980/J8FP75 (PMC10332559; doi:10.21980/J8FP75)

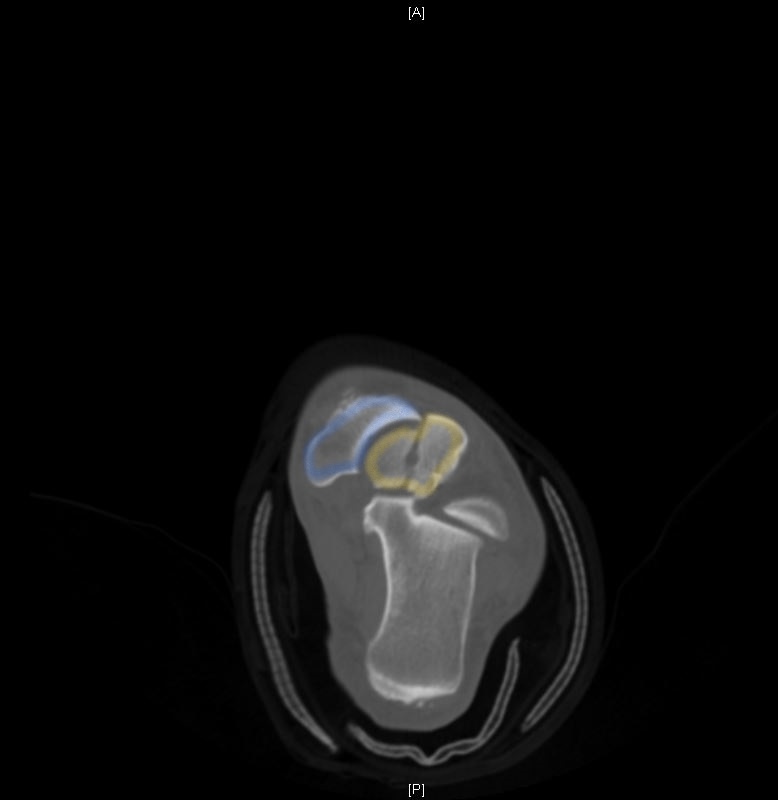

Supplement: Supplementary file 1 [file jetem-5-3-v7-supp1.jpg]

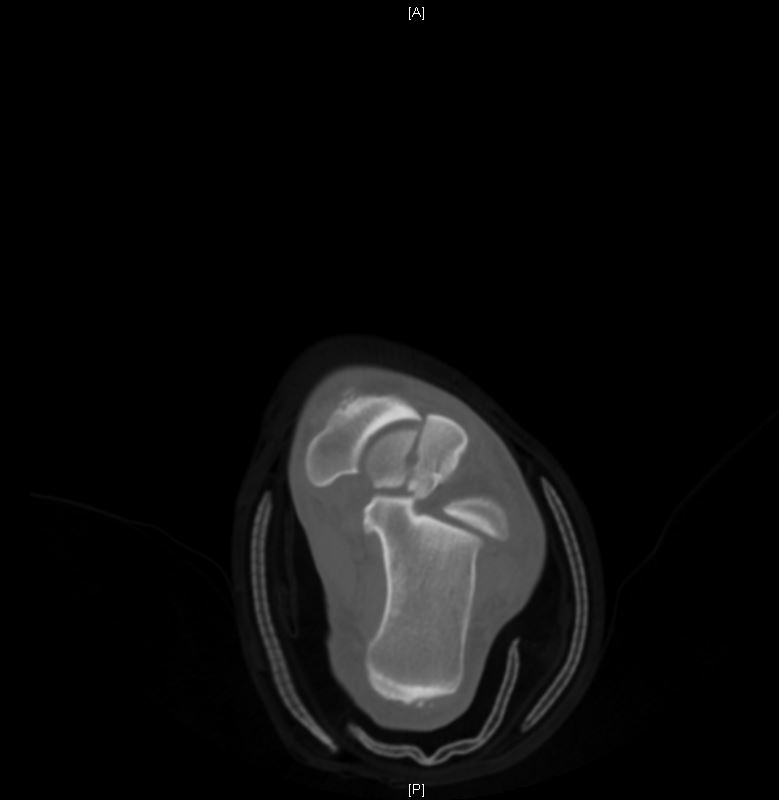

Supplement: Supplementary file 2 [file jetem-5-3-v7-supp2.jpg]

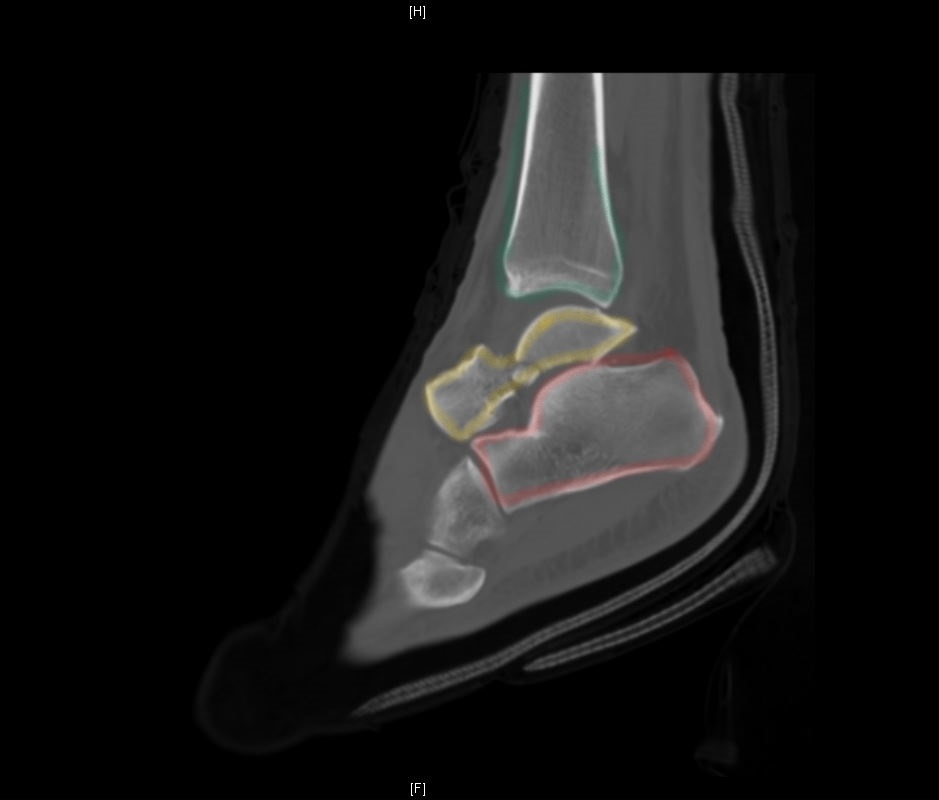

Supplement: Supplementary file 3 [file jetem-5-3-v7-supp3.jpg]

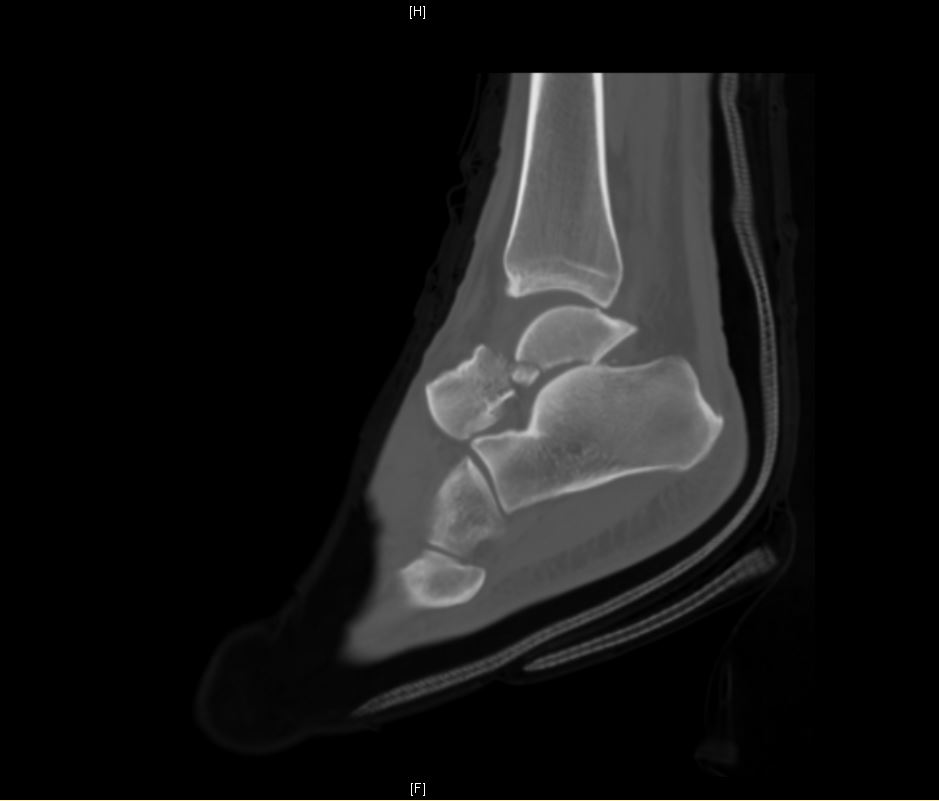

Supplement: Supplementary file 4 [file jetem-5-3-v7-supp4.jpg]

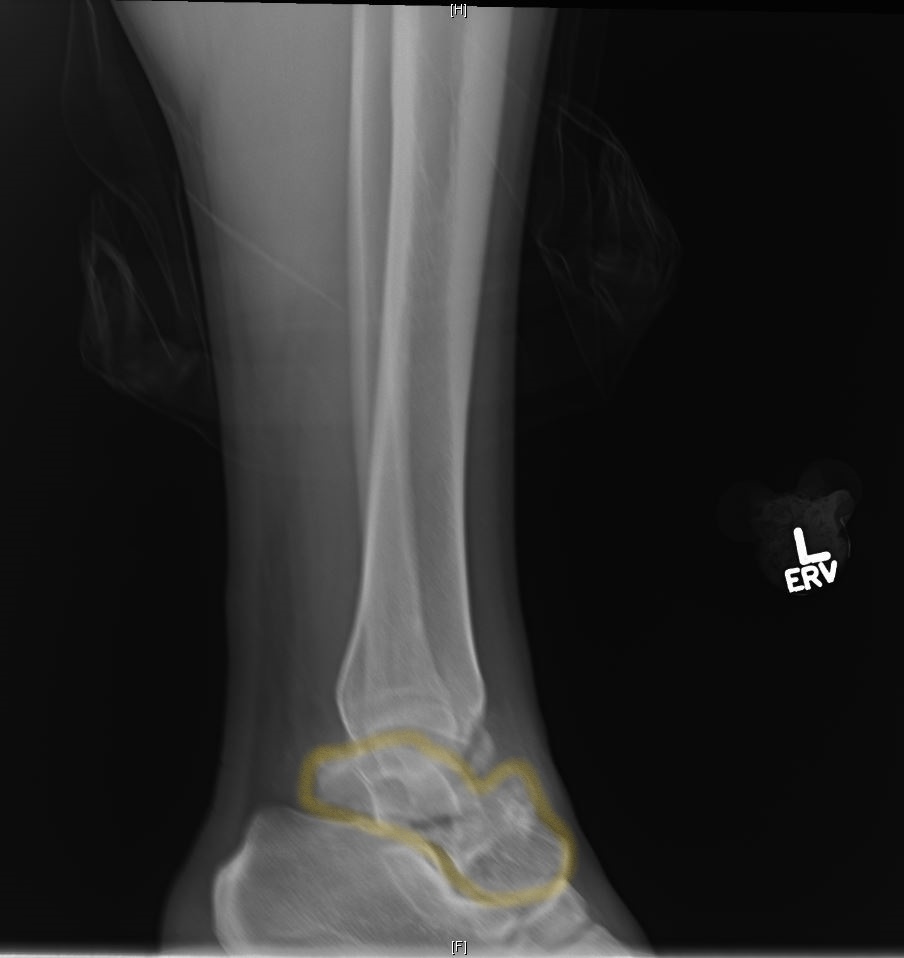

Supplement: Supplementary file 5 [file jetem-5-3-v7-supp5.jpg]

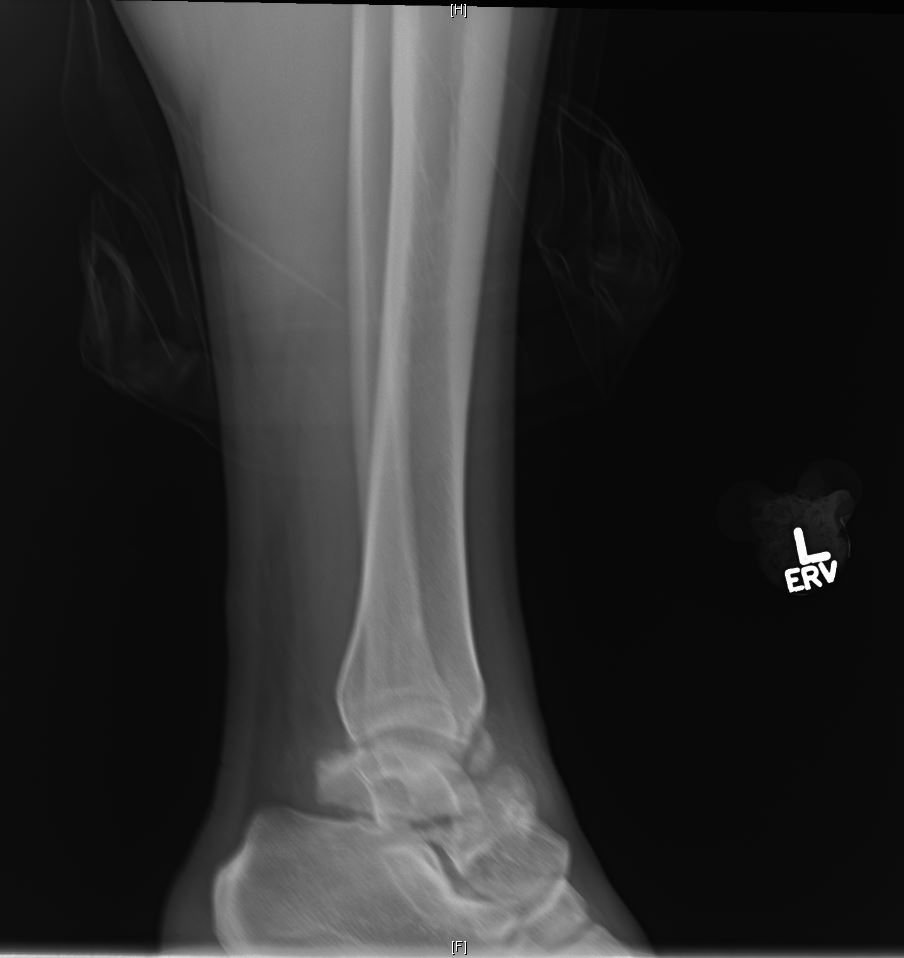

Supplement: Supplementary file 6 [file jetem-5-3-v7-supp6.jpg]
